# Noncompliance in people living with HIV: accuracy of defining characteristics of the nursing diagnosis^1^

**DOI:** 10.1590/1518-8345.1582.2940

**Published:** 2017-10-30

**Authors:** Richardson Augusto Rosendo da Silva, Mayara Mirna do Nascimento Costa, Vinicius Lino de Souza, Bárbara Coeli Oliveira da Silva, Cristiane da Silva Costa, Itaísa Fernandes Cardoso de Andrade

**Affiliations:** 2 PhD, Adjunct Professor, Departamento de Enfermagem, Universidade Federal do Rio Grande do Norte, Natal, RN, Brazil.; 3 MSc, RN, Hospital Maternidade Divino Amor, Prefeitura de Parnamirim, Parnamirim, RN, Brazil.; 4 Master’s student, Universidade Federal do Rio Grande do Norte, Natal, RN, Brazil. Scholarship holder at Coordenação de Aperfeiçoamento de Pessoal de Nível Superior (CAPES), Brazil.; 5 Master’s student, Universidade Federal do Rio Grande do Norte, Natal, RN, Brazil. Professor, Escola Técnica Potiguar, Universidade Potiguar, Natal, RN, Brazil.; 6 Undergraduate student in Nursing, Departamento de Enfermagem, Universidade Federal do Rio Grande do Norte, Natal, RN, Brazil. Scholarship holder at Conselho Nacional de Desenvolvimento Científico e Tecnológico (CNPq), Brazil.; 7 RN.

**Keywords:** Nursing Diagnosis, Validation Studies, Signs and Symptoms, Sensitivity and Specificity, Medication Adherence, Acquired Immunodeficiency Syndrome

## Abstract

**Objective:**

to evaluate the accuracy of the defining characteristics of the NANDA International nursing diagnosis, noncompliance, in people with HIV.

**Method:**

study of diagnostic accuracy, performed in two stages. In the first stage, 113 people with HIV from a hospital of infectious diseases in the Northeast of Brazil were assessed for identification of clinical indicators of noncompliance. In the second, the defining characteristics were evaluated by six specialist nurses, analyzing the presence or absence of the diagnosis. For accuracy of the clinical indicators, the specificity, sensitivity, predictive values and likelihood ratios were measured.

**Results:**

the presence of the noncompliance diagnosis was shown in 69% (n=78) of people with HIV. The most sensitive indicator was, missing of appointments (OR: 28.93, 95% CI: 1.112-2.126, p = 0.002). On the other hand, nonadherence behavior (OR: 15.00, 95% CI: 1.829-3.981, p = 0.001) and failure to meet outcomes (OR: 13.41; 95% CI: 1.272-2.508; P = 0.003) achieved higher specificity.

**Conclusion:**

the most accurate defining characteristics were nonadherence behavior, missing of appointments, and failure to meet outcomes. Thus, in the presence of these, the nurse can identify, with greater security, the diagnosis studied.

## Introduction

In the early 1990s, an important milestone in the treatment of Acquired Immunodeficiency Syndrome (AIDS) was the development of high-potency combined antiretroviral therapy^1^. The great benefit generated by the use of this therapy was the prolongation of the survival of people who acquired this disease, since it ceased to be considered fatal, but rather a chronic condition^2^.

Despite the improvements generated by this therapy, many difficulties must be overcome. One of them is the patient’s lack of adherence to treatment, which brings challenges to care and to health professionals. Thus, adherence is a dynamic and multifactorial process that encompasses physical, psychological, social, cultural and behavioral aspects, requiring shared decision-making between the person with HIV, the health team, and the social network^2^ ^-^ ^3^.

The follow-up should be careful, planned and recorded. It is not only a matter of including the classic questions, “Are you taking everything correctly?”, but to investigate in detail the routine adopted by the patient to take the medication, difficulties encountered, and failures^3^.

The lack of adherence behavior is associated with the fact that the individual does not engage adequately in recommended behaviors, and/or engages in unhealthy behaviors, and/or does not show an interest/effort to follow professional recommendations or to acquire knowledge about the disease. Thus, noncompliance is considered a threat to the effectiveness of treatment at the individual level, and to the spread of virus resistance at the collective level, increasing cases of HIV infectivity^3^ ^-^ ^4^.

Noncompliance (code 00079) is a nursing diagnosis (ND), categorized within Taxonomy II of NANDA-I, which consists of a classification system of NDs accepted as representations of nursing knowledge to support the profession, by means of clinically useful terminology^4^.

The noncompliance diagnosis (code 00079) is included in the Domain 1 - Health promotion, Class 2 - Health management. Although the diagnoses proposed by this taxonomy are well recognized and used for different situations and scenarios, they are not definitive, because research in specific populations, by means of clinical validation studies, allow for their improvement^4^ ^-^ ^5^.

Thus, the clinical validation process of the NDs is relevant for clinical management and for implementation of systematic practices, as the diagnostic accuracy process establishes, with greater accuracy, the defining characteristics that predict the proximity to the diagnosis, among many others. The accuracy of a ND is defined as the evaluator’s judgment as to the degree of relevance, specificity and consistency of the clues (clinical indicators) available for diagnosis^6^.

For analysis of the accuracy of each defining characteristic, mathematical calculations are performed, with emphasis on the following dimensions: sensitivity, specificity, positive and negative predictive values. Through diagnostic accuracy measures, it is possible to differentiate individuals with and without the ND, from the defining characteristics. However, it is well known that the stimulation of the development of research in this field reduces the nurses’ subjective uncertainties and leads to more simplified diagnosis^6^.

One study of accuracy of the diagnosis, *impaired gas exchange* (code 00030) in children with acute respiratory infection, showed that conducting research demonstrating the validity of the relationships between the clinical manifestations and the ND corroborates the reduction of inaccuracy in choosing the diagnosis, by discriminating the most appropriate ND to situations/problems presented by the patient and, consequently, achieving positive health outcomes^7^.

This study intended to corroborate the accuracy of clinical evidence for such diagnoses, as well as to contribute to the process of diagnostic inference, increasing the nurse’s diagnostic ability, reducing clinical uncertainties, so that actions and nursing interventions can be initiated with scientific support. Therefore, the objective of this study was to evaluate the accuracy of the defining characteristics of the NANDA-I diagnosis, *noncompliance*, in people with HIV.

## Method

**Figure 1:**
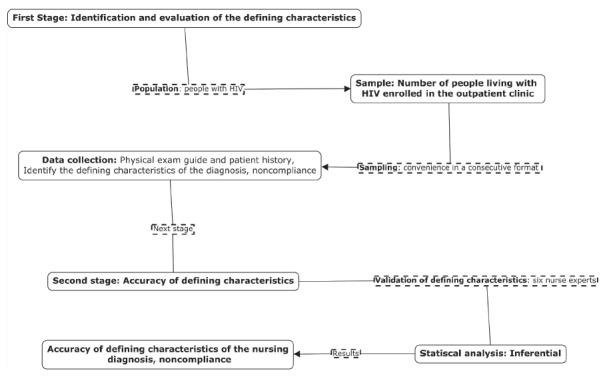
Methodological course for the accuracy of the defining characteristics of the nursing diagnosis, *noncompliance*

This was a diagnostic accuracy study, with a cross-sectional design, performed in two stages. In the first, the identification and evaluation of the NANDA-I clinical indicators of *lack of adherence* to antiretroviral treatment in people living with HIV were performed. In the second stage, the accuracy of the defining characteristics of the respective diagnosis was developed. The information in question is detailed in Figure 1, below.

In the first step, the population of people with HIV was from the outpatient HIV clinic of an intermunicipal referral hospital for the treatment of infectious diseases in the Northeast of Brazil. The fact that the institution cares for patients from all over the state, plus the distance from some cities, makes access difficult, and explains the non-attendance of some patients to the scheduled appointments. The institution is public in nature, with a mean of 800.2 visits per year of people with HIV, with or without complications; this includes ambulatory care, urgent care, specialized care service (SCS), and intensive care.

The flow of care begins at the outpatient clinic by spontaneous demand, or when referred by other services, with a medical and nursing appointment, and tests such as viral load, CD4 and CD8, so that the therapeutic scheme can be implemented, with the receipt of medications from the pharmacy.

Specifically in the outpatient clinic where patients are receiving antiretroviral treatment, 306 patients are registered and followed. Of this total, 158 patients did not get medication at the pharmacy and did not attend the appointment in 2014, corresponding to 51.63%. In this sense, this population was considered as the universe for the sample calculation. Thus, the formula for finite populations was used, and the criteria adopted were a 95% confidence level (Z∞ = 1.96) and a sample error of 5%, resulting in a sample size of 113 patients^8^.

It is noteworthy that, in the service, there is a record of patients in the outpatient clinic who, although they attend their scheduled appointments, have low adherence to medications.

Recruitment was by convenience in a consecutive manner, adopting the following inclusion criteria: clinically diagnosed with AIDS, age above 18 years, included in the hospital unit system as a low-adherence patient, using antiretroviral therapy for at least six months, and registered at the hospital outpatient clinic at the time of data collection. The exclusion criterion was presenting with altered physical or emotional conditions during the period of data collection that could interfere with such collection.

The data collection occurred from July 15 to September 30, 2015, by means of a physical examination and patient history guide, prepared according an integrative review conducted in national and international journals^4^ ^-^ ^7^, and complimentary books^9^ as well as the manual of the Ministry of Health (MS)^10^, subdivided into two parts.

The first one contains variables such as sex, age group, level of education, monthly income, form of contamination, and time of diagnosis. The second one is focused on questions regarding medication adherence, such as: length of time the medication is used, number of hospitalizations, missing appointments, adverse effects after medication, receiving medication, signs of worsening disease, illness and treatment, team/patient relationship, satisfaction with the service, access to the service, health beliefs, motivational forces, social and family support, life habits, health beliefs incompatible with the therapeutic regimen, expectations regarding treatment, ability to implement the therapeutic regime, cultural incompatibility and spiritual values incompatible with the plan.

With these data, the defining characteristics of *noncompliance* were measured, such as: developmental-related complication, nonadherence behavior, exacerbation of symptoms, failure to meet outcomes, and missing of appointments.

*Developmental-related complication* is defined as signs and symptoms indicative of the onset of complications of the disease, due to failure to adhere to treatment. *Nonadherence behavior* is associated with the fact that the individual does not engage appropriately in recommended behaviors, engages in unhealthy behaviors, and/or shows no interest/effort in following professional recommendations or acquiring knowledge about the disease. *Exacerbation of symptoms* demonstrates the damage to disease control. *Failure to meet outcomes* is defined as not achieving the expected benefits after instituting pharmacological treatment. *Missing of appointments* is the difficulty of the individual to attend scheduled appointments and/or have difficulties in adopting and maintaining healthy lifestyle habits^11^.

After development of the instrument, the content was submitted to a process of normalization and validation by eight nurses, professors and specialists in Systematization of Nursing Care and immunology. The suggestions were included and it was reassessed again. After that time, 10% of the sample was used for a pretest, so that possible gaps could be identified, with no need for modifications identified. The data collection was performed by two-master’s students and one doctoral student, who participated in a ten-hour course, divided into two days, by the researcher in charge. On the first day, subjects related to antiretroviral medications were discussed, such as the type of medication, adverse reactions, and *noncompliance*. In the second, the instrument was presented, and the approach to the person with HIV was simulated.

Data collection occurred in a reserved room in the hospital, in order to maintain the privacy of the participants and avoid interruptions. Then, with the data obtained from 113 people with HIV, the researchers cataloged the defining characteristics of the diagnosis, based on clinical, social, and behavioral evidence.

The second step of the process returned to the content validation proposed by Fehring in its entirety. This model consists of validation of the defining characteristics of the diagnosis by experts, whose selection of specialists must be by established criteria. Thus, the validation process goes through several stages, namely: selection of specialists; assigning a value to the defining characteristic of the ND; Delphi technique (optional); weighted mean of the assigned scores; and, finally, inferential analysis of the data^6^.

The author recommends that expert nurses perform this type of study. However, the difficulties in finding a sample of professionals that meet the criteria proposed by the author are known^6^, and the author recognizes this fact. Thus, the criteria were modified for this study, and described as follows: (1 point) master’s degree in nursing; (2 points) master’s degree in nursing with dissertation related to contents relevant to the diagnosis under study; (1 point) publication of an article on ND associated with validation methods in reference journals; (2 points) article published on ND with content relevant to the area in focus; (4 points) doctoral degree dealing with ND; (1 point) clinical experience of at least one year in the area of the diagnosis under study; (2 points) certificate of clinical practice relevant to the area of the diagnosis under study.

After searching for specialists, six were selected, in the area of ND, with clinical practice or experience in the teaching of infectious diseases in nursing. Then, theoretical and practical training was given to the specialists, with the application of realistic situations, so that their responses were measured based on capacity, efficiency, and trend, false positive and negative rates, as described in Table 1.

**Table 1:** Cut-off points for evaluation of diagnostic inference ability, in a city in the Northeast Region. Brazil, 2015

**Parameters**	**Acceptable**	**Marginal**	**Unacceptable**
**Efficacy (E)**	**0.9 or more**	**>0.8-0.9**	**Less than 0.8**
**False positive rate (FP)**	**0.05 or less**	**≤0.10**	**More than 0.10**
**False negative rate (FN)**	**0.02 or less**	**≤0.10**	**More than 0.10**
**Trend (T)**	**0.80-1.20**	**0.50-0.80 or 1.2-1.5**	**Less than 0.50 or more than 1.5**

The practice of training was fundamental because there is no gold standard for the identification of NDs. In this study, the characteristics observed in patients who showed adherence for taking the medication (no delay in receiving the drug, CD4 lymphocyte count greater than 200 cells/mm^3^, undetectable viral load, absence of opportunistic diseases, and not reporting difficulties in taking medications) and attending appointments.

For the analysis of diagnostic inference ability, an instrument developed by the researchers was used, consisting of 12 items that related to each item to be evaluated, resulting in a mean that was compared with the acceptable, marginal and unacceptable levels. After analyzing the results, only three specialists were able to obtain the acceptable mean (> 0.9) for each item of diagnostic ability assessment.

After selecting the specialists, a spreadsheet was prepared for each patient in Microsoft Office Excel, with the possible defining characteristics, totaling 113, and a column with the scale composed of the following values: 1 = not at all relevant; 2 = a little relevant; 3 = relevant; 4 = very relevant; 5 = very much relevant, if it was part of the diagnosis. The spreadsheets were sent to the specialists, together with the synthesis of each case, so that they had a full perception of the health conditions of people with HIV and performed the inferential data analysis.

After the specialist nurses assigned value to each of the DCs, a mean of the scores assigned to each of the DCs was calculated, with the following weights being assigned to the calculations: weight 1 = 0, weight 2 = 0.25, weight 3 = 0.50, weight 4 = 0.75, weight 5 = 1. Then, DCs with a weighted mean ≤0.50 were discarded, and DCs with a weighted mean ≥0.80 were considered as the primary ones. Finally, the total diagnostic content validation score was obtained by summing the individual scores; divided by the total number of DCs, excluding those with a weighted mean ≤0.50.

Descriptive and inferential statistics were used for data analysis, using the IBM Statistical Package for the Social Sciences (SPSS), version 20.0, for Windows. The accuracy of the DCs of *noncompliance* was evaluated using sensitivity, specificity, negative and positive predictive values. A cut-off point of 80% was used for the DCs, and results obtained above that point were considered relevant. The reason why the researchers adopted such a cut-off point is based on other studies of accuracy in which the same value was used, showing that this cut-off point conferred greater statistical representativeness ^(^ ^12^ ^-^ ^13^. Thus, the positive and negative likelihood ratio, diagnostic odds ratio (DOR) and prevalence were calculated.

The research was approved by the Research Ethics Committee of the institution, Protocol No. 1,146,907, obtaining approval with Certificate of Presentation for Ethical Assessment (CAAE) nº 46206215.3.0000.5537.

## Results

A total of 113 people with HIV were evaluated: 55.5% of whom were heterosexual (66.4%); 39% were 30 to 39 years of age; 55.7% had not completed elementary school; and 47.8% had an income of minimum wage (n = 54). Regarding the time of AIDS diagnosis, the mean was five years (± 5.38). Regarding the presence of the *noncompliance* diagnosis, 69% (n = 78) of people with HIV had the diagnosis. In addition, Table 2 shows the prevalence ratio among the DCs, in which is possible to observe that all associations were statistically significant.

**Table 2:** Prevalence reasons for defining characteristics, according to the occurrence of the NANDA-I nursing diagnosis, *noncompliance*, in people with HIV, in a municipality in the Northeast Region. Brazil, 2015

**Variables**		**Nursing diagnosis of noncompliance**					**Statistics**			
**Defining characteristics**		**Present**			**Absent**			**Total**		
		**N**	**%**		**N**	**%**		**N**	**%**	
**Nonadherence behavior**										
	**Present**	**78**	**69.03**		**00**	**00.00**		**78**	**69.03**	**p=0.001** ^*^
	**Absent**	**00**	**00.00**		**35**	**30.97**		**35**	**30.97**	**PR** ^**†**^ **=3.582** ^**†**^
	**Total**	**78**	**69.03**		**35**	**30.97**		**113**	**100**	**CI** ^**‡**^ **95%: 1.829-3.981** ^**‡**^
**Development-related complication**										
	**Present**	**58**	**51.33**		**10**	**8.85**		**68**	**60.18**	**p=0.04** ^**§**^
	**Absent**	**20**	**17.70**		**25**	**22.12**		**45**	**39.82**	**PR** ^**†**^ **=2.671** ^**†**^
	**Total**	**78**	**69.03**		**35**	**30.97**		**113**	**100**	**CI** ^**‡**^ **95%: 1.729-2.873** ^**‡**^
**Exacerbation of symptoms**										
	**Present**	**48**	**42.48**		**17**	**15.04**		**65**	**57.52**	**p=0.03** ^**§**^
	**Absent**	**30**	**26.55**		**18**	**15.93**		**48**	**42.48**	**PR** ^**†**^ **=1.95** ^**†**^
	**Total**	**78**	**69.03**		**35**	**30.97**		**113**	**100**	**CI** ^**‡**^ **95%: 1.152-2.607** ^**‡**^
**Failure to meet outcomes**										
	**Present**	**69**	**61.06**		**04**	**3.54**		**73**	**64.60**	**p=0.003** ^*^
	**Absent**	**09**	**7.97**		**31**	**27.43**		**40**	**35.40**	**PR** ^**†**^ **=1.872** ^**†**^
	**Total**	**78**	**69.03**		**35**	**30.97**		**113**	**100**	**CI** ^**‡**^ **95%: 1.272-2.508** ^**‡**^
**Missing of appointments**										
	**Present**	**73**	**64.60**		**03**	**2.65**		**76**	**67.25**	**p=0.002** ^*^
	**Absent**	**05**	**4.43**		**32**	**28.32**		**37**	**32.75**	**PR** ^**†**^ **=1.351** ^**†**^
	**Total**	**78**	**69.03**		**35**	**30.97**		**113**	**100**	**CI** ^**‡**^ **95%: 1.112-2.126** ^**‡**^

*Fisher exact test; †PR=prevalence ratio; ‡CI=confidence interval of 95%; §Chi-square test Pearson; p<0,05

Regarding the accuracy of the defining characteristics, *missing of appointments* (96.21%) presented the greatest sensitivity, in which values of likelihood and Diagnostic Odds Ratio (DOR) were statistically significant, as shown in Table 3.

**Table 3:** Measures of accuracy of the defining characteristics of the nursing diagnosis, *noncompliance*, in people with HIV, in a municipality of the Northeast Region. Brazil, 2015

**Defining characteristics**	**Se** ^*^	**Sp** ^**†**^	**PPV** ^**‡**^	**NPV** ^**§**^	**PLR** ^**||**^ **(IC 95%)**	**NLR** ^**¶**^ **(IC 95%)**	**DOR** ^**^ **(IC 95%)**
**Development-related complication**	**57.00**	**81.74**	**94.13**	**36.87**	**2.00** **(0.97-2.61)**	**0.70** **(0.41-1.61)**	**3.00** **(0.94-4.58)**
**Nonadherence behavior**	**64.30**	**95.24**	**98.41**	**49.56**	**7.00** **(1.32-8.12)**	**0.62** **(0.41-0.67)**	**15.00** **(8.16-17.74)**
**Missing of appointments**	**96.21**	**70.84**	**93.45**	**65.34**	**4.33** **(1.58-5.04)**	**0.20** **(0.09-0.24)**	**28.93** **(9.87-32.85)**
**Exacerbation of symptoms**	**88.87**	**68.66**	**87.84**	**42.55**	**1.46** **(0.73-2.91)**	**0.76** **(0.52-1.13)**	**1.90** **(0.65-5.56)**
**Failure to meet outcomes**	**53.26**	**84.55**	**97.50**	**48.44**	**9.46** **(1.25-10.29)**	**0.65** **(0.53-0.70)**	**13.41** **(1.95-14.31)**

*Se=sensibility; †Sp=specificity; ‡PPV= positive predictive value; §NPV= negative predictive value; ||PLR= Positive likelihood ratio; ¶NLR= Negative likelihood ratio; **DOR=Diagnostic *Odds Ratio*

Among the five defining characteristics, three obtained a cut-off point above 80%, being specific to the diagnosis of *noncompliance*, namely: *nonadherence behavior*, *failure to meet outcomes* and *development-related complication*. However, only the first two showed statistically significant likelihood and DOR values. The DCs that presented statistical significance were tested by means of logistic regression, to verify the conjugated association of the DCs, as revealed in Table 4.

**Table 4:** Logistic regression for the predictive characteristics of the presence of the nursing diagnosis, *noncompliance*, in people with HIV, in a municipality in the Northeast Region. Brazil, 2015

**Defining characteristics**	**Se** ^*^	**SP** ^**†**^	**PPV** ^**‡**^	**NPV** ^**§**^	**PLR** ^**||**^ **(IC 95%)**	**NLR** ^**¶**^ **(IC 95%)**	**DOR** ^**^ **(IC 95%)**
**Development-related complication**	**57.00**	**81.74**	**94.13**	**36.87**	**2.00** **(0.97-2.61)**	**0.70** **(0.41-1.61)**	**3.00** **(0.94-4.58)**
**Nonadherence behavior**	**64.30**	**95.24**	**98.41**	**49.56**	**7.00** **(1.32-8.12)**	**0.62** **(0.41-0.67)**	**15.00** **(8.16-17.74)**
**Missing of appointments**	**96.21**	**70.84**	**93.45**	**65.34**	**4.33** **(1.58-5.04)**	**0.20** **(0.09-0.24)**	**28.93** **(9.87-32.85)**
**Exacerbation of symptoms**	**88.87**	**68.66**	**87.84**	**42.55**	**1.46** **(0.73-2.91)**	**0.76** **(0.52-1.13)**	**1.90** **(0.65-5.56)**
**Failure to meet outcomes**	**53.26**	**84.55**	**97.50**	**48.44**	**9.46** **(1.25-10.29)**	**0.65** **(0.53-0.70)**	**13.41** **(1.95-14.31)**

* Se=sensibility; †Sp=specificity; ‡PPV= positive predictive value; §NPV= negative predictive value; ||PLR= Positive likelihood ratio; ¶NLR= Negative likelihood ratio; **DOR=Diagnostic *Odds Ratio*

## Discussion

The clinical validation process of the NDs, performed by obtaining accuracy in the DCs, is a practice that corroborates the process of differentiation of the presence and absence of diagnoses, as well as attenuating the biases of the clinical inferences of the nurse, as the interpretation of the clinical evidence is subjective^6^ ^-^ ^7^.

In the study, the *noncompliance* diagnosis is defined as the “behavior of the person and/or caregiver that fails to coincide with a health-promoting or therapeutic plan agreed on by the person (and/or family, and/or community) and health care professional. In the presence of an agreed--upon, health-promoting or therapeutic plan, the person’s or caregiver behavior is fully or partly nonadherent and may lead to clinically ineffective or partially effective outcomes”^9^. This diagnosis is presented with the DCs, *development-related complication*, *nonadherence behavior*, *exacerbation of symptoms*, *failure to meet outcomes* and *missing of appointments* ^9^.

Among these, about 67.3% of the patients with a *noncompliance* diagnosis had the DC, *missing of appointments*; that is, they did not attend scheduled appointments, did not take doses of indicated medications, did not take medication at the correct time and did not attend the health service to receive the medication^14^.

In this sense, the non-attendance at the care site may be aimed at not accepting the new health condition and a disbelief in the medication regimen^14^. The complexity of treatment requires the person with HIV to have previous guidelines, because some medications need to be taken with food, others while fasting, or in temporal sequences combined with other medications, which requires the patient’s organization and commitment to treatment^15^.

Studies demonstrate that the adherence to antiretroviral therapy can also be influenced by the reduction of barriers to accessing health services; use of health technologies, such as welcoming, bonding, accountability, and trust in the service; and, improvement of the interpersonal relationships with health professionals^1^ ^-^ ^4^.

The role of the professional who provides care is important, especially his/her ability for dialogue and negotiation. Guidance on the disease, the importance of adherence, medications and the correct way of using them, the side effects of the treatment, the actions to be taken, and when these effects occur, are a priority in the treatment and should involve all the professionals in the health care services^2^ ^-^ ^4^.

In the present study, in addition to the respective factors of abstention, the missing of appointments was also related to forgetfulness. In a study showing that forgetting was the main cause for missing appointments, health professionals implemented telephone follow-up, considerably reducing the number of missed appointments^15^. Thus, nurses can implement active searches and therapeutic activities, such as conversation wheels and community therapies^2^ ^,^ ^16^ ^-^ ^17^.

The second DC, *development-related complication*, was identified in the *noncompliance* diagnosis, which had as indicators the signs and symptoms presented by the evolution of the disease and its complication, most often due to failure to adhere to treatment^9^ ^-^ ^17^.

Researchers point out that one of the motives claimed by people with HIV for non-adherence is that they are not aware of the importance of antiretroviral medications, suppression of viral load, increasing the CD4 and CD8^18^, and attenuation of the development-related complications and opportunistic infections, which are linked to abandonment or irregularity in the continuity of treatment^18^. Therefore, the nurse can implement actions that corroborate the demystification of medications regarding their use and side effects^2^ ^,^ ^16^ ^-^ ^17^.

In order to help patients to improve their adherence to antiretroviral treatment, many strategies have been described in the literature. Some of these must be implemented with rigor, such as the formation of more effective membership groups or individual care groups that respond to the expectations and concerns of medications, providing free reminders, time tables, mobile apps, as well as medication diaries^1^ ^-^ ^4^ ^,^ ^14^. In the research scenario, strategies are adopted such as support groups for adherence, and the provision of guidance books and journals for recording the use of antiretroviral.

In another study, a home-based therapeutic care program was adopted, and showed that patients of that program had significantly greater adherence compared to conventional outpatient treatment^17^. Likewise, in practice, different strategies can be used to promote adherence; however, it is important that the development and implementation of these interventions be realistically designed for specific groups, taking into account individual characteristics, style of life, and social support^14^.

Another characteristic, considered specific to the diagnosis studied, was *nonadherence behavior*, in which the individual shows no interest in following the professional recommendations about the treatment, nor in acquiring knowledge about the disease, in addition to practicing unhealthy behaviors^19^.

Studies present reports of patients who demonstrate *nonadherence behavior*, such as lack of interest in learning about the illness and treatment related to depression, aggression, denial of illness, feeling of revulsion, as well as an unhealthy lifestyle with alcohol and drug abuse^18^ ^-^ ^20^.

Another study demonstrated that religion also contributes to *nonadherence behavior*, in which people with HIV have religious beliefs and faith that they will be cured by a divine entity, as their reason for failure to follow medication treatment^21^. A study indicated that the excessive rhythm of work, resulting in lack of time to take medications, frequent trips, not eating at the correct times, leaving home without the medication, and the amount of medication to take per day were factors that contributed to *nonadherence behavior* ^22^ ^-^ ^23^.

Regarding the more specific indicators for the determination of the *noncompliance* diagnosis, *exacerbation of symptoms* and *failure to meet outcomes* were identified. In research, the majority of patients experience challenges at the beginning of treatment, contributing to the *failure to meet outcomes*. These occur due to the initial reactions caused by the use of antiretroviral, such as nausea, vomiting, diarrhea, gas, heartburn, drowsiness, insomnia, and nightmares, among other issues^23^ ^-^ ^24^.

*Failure to meet outcomes* is presented as not achieving the expected benefits after the institution of medication treatment. Therapeutic success with highly active antiretroviral therapy (HAART) is demonstrated by an undetectable viral load, immune reconstitution confirmed by CD4+T lymphocytes, the absence of clinical manifestations, and absence of exacerbation of disease symptoms. Adherence to treatment has been consistently associated with achieving therapeutic success and this, in turn, may enhance the adherence. It was pointed out in a study that showed that patients with a CD4 count lower than 200 cells/mm^3^ presented a higher risk of *noncompliance* ^25^.

It is important to highlight that the analysis of these clinical indicators, in an isolated way, seeks to better understand the specificities that interfere in the formulation of the diagnosis, as well as the nurse’s knowledge^3^ ^-^ ^6^, such as the fact that antiretroviral therapy (ART) delays the development of the disease and reduces the exacerbation of symptoms, by means of viral suppression and immune system restoration. Thus, it is important to discuss and explain the results of the laboratory tests to the patient during the appointments, so that he understands and visualizes his progress, recognizing the advantages of the treatment, thus strengthening the motivation for adherence^16^ ^-^ ^25^.

Given this, it is understood that *noncompliance* is, in turn, one of the main problems of people with HIV, as it is a chronic disease, which, by itself, represents one more difficulty for following the recommendations. However, early identification and greater safety by the nurse can be performed through the use of accurate clinical indicators, directed interventions, and nursing outcomes with greater effectiveness^25^.

Although many strategies are still needed to end the AIDS epidemic, there is an explicit recommendation for the provision and early beginning of antiretroviral therapy as soon as the person is diagnosed with HIV (Treatment as Prevention - TASP). In addition, it is important to highlight the relevance of the *noncompliance* diagnosis, from the perspective of the cascade of care, proposed internationally, in which adherence represents a fundamental stage, including for reaching the goals 90-90-90, i.e., that by the year 2020: 90% of all people with HIV will know they have the virus; 90% of all people with diagnosed HIV infection will receive antiretroviral therapy without interruption; and, 90% of all people receiving antiretroviral therapy will obtain viral suppression.

Finally, it is important to note that, despite the relevance of accuracy studies, these are recent and remain scarce in the nursing area^6^. In addition, the lack of studies that also analyze accuracy in NDs related to adherence, both involving HIV and other chronic conditions, is limited to the reliability of inferences made by nurses to accurately identify the presence of the diagnosis.

However, in the case of other chronic diseases, a study evaluating the accuracy of the DCs of the nursing diagnoses *excess fluid volume* (code 00026) in patients with chronic renal failure, indicated that the DCs identified as accurate can help nurses in the inference process of this diagnosis in patients undergoing hemodialysis, allowing greater safety in the choice of diagnosis, as well as the selection of outcomes and interventions with greater chances of targeting and efficacy^12^.

One study investigating the accuracy of the DCs of the diagnosis of *ineffective family therapeutic health management* (code 00080) in diabetic patients receiving care at a family health center, showed the need to work on adherence from a family perspective, especially considering the complexity of treatment for people with chronic disease^13^.

As a limitation of the research, we highlight the difficulty in characterizing the conceptual and operational definitions of the DCs related to the diagnosis studied, which were not clearly defined in the literature: an obstacle that may impair the reliability of the data collected, regarding the measurement. In this way, it is also proposed to investigate, in future research, the validation of the conceptual and operational definitions of the DCs of the *noncompliance* diagnosis.

## Conclusion

The present data demonstrate that the DC with higher sensitivity was *missing of appointments* due to the *noncompliance* diagnosis. As for the higher specificity, *nonadherence behavior* and *failure to meet outcomes* were highlighted. In addition, the indicators, *nonadherence behavior, missing of appointments* and *failure to meet outcomes* were identified in the logistic regression model as the most accurate DCs for identifying the diagnosis in people with HIV.

The DCs studied will help the nurse in inferring the nursing diagnosis of *noncompliance* in people with HIV, with greater accuracy and safety, and thus enable them to establish nursing outcomes and interventions with greater chances of targeting and effectiveness.
